# Should we pay attention to surgeon or hospital volume in total knee arthroplasty? Evidence from a nationwide population-based study

**DOI:** 10.1371/journal.pone.0216667

**Published:** 2019-05-10

**Authors:** Tsung-Hsien Yu, Ying-Yi Chou, Yu-Chi Tung

**Affiliations:** 1 Department of Health Care Management, National Taipei University of Nursing and Health Sciences, Taipei, Taiwan; 2 Institute of Health Policy and Management, National Taiwan University, Taipei, Taiwan; Cleveland Clinic, UNITED STATES

## Abstract

**Background:**

Although prior research into the relationship between volume and outcome indicates that this relationship is not linear and that an optimal volume should be specified, consensus is lacking regarding the ideal value of this optimal volume. The purposes of this study were to use a visual method to identify surgeon- and hospital-volume thresholds and to examine the relationships of surgeon and hospital volume thresholds to 30-day readmission.

**Methods:**

A retrospective nationwide population-based study design was adopted. Patients who received total knee replacement surgery between 2007 and 2008 in any hospital in Taiwan were included. After adjusting for patient, physician, and hospital characteristics, a restricted cubic spline regression model was used to identify optimal surgeon- and hospital-volume thresholds. Further, a patient-level mixed effect model was conducted to test the respective relationships between these thresholds and 30-day readmission.

**Results:**

A total of 30,828 patients who had received their surgeries from 1,468 surgeons in 437 hospitals were included in this study. Thresholds of 50 cases a year for surgeons and 75 cases a year for hospitals were identified using a restricted cubic spline regression model. However, only the surgeon volume threshold was associated with 30-day readmission using a patient-level mixed effect model after adjusting for patient-, surgeon- and hospital-level covariates.

**Conclusions:**

According to the results of the restricted cubic spline models, the optimal volume thresholds for surgeons and hospitals are 50 cases and 75 cases a year, respectively. However, only the surgeon volume threshold is associated with 30-day readmission.

## Introduction

The relationship between healthcare provider volume and outcomes has been extensively studied since Luft *et al* published their foundational article in 1979 [1], which spurred research interest in the volume-outcome relationship and triggered further research on various surgical operations and medical conditions such as orthopedic surgeries [2], coronary artery bypass grafts (CABGs) [3, 4], acute myocardial infarction [5], stroke [6], and cancer [7, 8]. Moreover, the findings of these and other related studies have been applied by stakeholders for various purposes [9–13]. Although volume-outcome research supports that the volume-outcome relationship is not linear and that there should be an optimal volume, consensus regarding the value of this optimal volume has yet to emerge.

The need to set an optimal volume standard is grounded in the well-established association between low volume and worse patient outcomes. Lau *et al* reviewed 11 volume-mortality studies for total knee arthroplasty (TKA) and found that the suggested optimal volume standard ranges from 3 to 52 operations a year for surgeons [14] and from 25 to 110 operations a year for hospitals [15, 16]. Studies in the literature have largely adopted one of two major approaches to define the low-volume threshold, including expert consensus [17–19] and equality distribution [20, 21]. The former relies on expert experience and opinion, so the representative of attendees may influence the cutoff-point selection. The latter uses statistical methods to divide provider volume into groups such as quartile [20, 22] and quintile [23–26] to determine a low-volume threshold. However, the distribution of service volume that is used in this approach may be skewed [22]. Based on their drawbacks, the appropriateness of using these two approaches should be reevaluated. Besides, the heterogeneity of determining low volume may produce different results [27, 28]. Therefore, a standardized and visual method of determining the low-volume threshold is needed [29].

This study used TKA as its example and employed a visual method to identify surgeon and hospital volume thresholds. Furthermore, we examined the relationships of between surgeon and hospital volume thresholds to outcome of care.

## Methods

### Study design

This retrospective nationwide population-based study design was adopted to examine the relationship between provider volume and 30-day readmission after adjusting for patient-, physician-, and hospital-level covariates.

### Data source

Data for this study was obtained from the Taiwan National Health Insurance Research Database (NHIRD). The NHIRD, published by the Taiwan National Health Research Institute, includes all of the original claims data and registration files for beneficiaries enrolled under the National Health Insurance (NHI) program. The database covers the 23 million Taiwanese enrollees (approximately 98% of the population) in the NHI program. In addition, the NHIRD is a de-identified secondary database containing patient-level demographics and administrative information. The data are released for research purposes. The protocol for this study was approved by the Institutional Review Board of the National Taiwan University Hospital (protocol #201408005W) on 12th August 2014.

### Study population

The study included all patients who received TKA surgery (International Classification of Diseases, Ninth Revision, Clinical Modification [ICD-9-CM] procedure code: 81.54) between 2007 and 2008 at any hospital in Taiwan. Patients who were under 18 years (n = 20) of age or who had received TKA surgery 3 months or more before the index hospitalization event (n = 1,035) were excluded in order to restrict our evaluation to an adult population and to avoid misclassifications of readmission. In addition, patients whose surgeon’s age or seniority was unknown (n = 33) were excluded.

### Definition of variables

Readmission within 30 days of discharge for total knee replacement was used as the measure of outcome of care because this indicator is commonly used in the literature and practice [30–32]. Surgeon and hospital volumes were used as independent variables, defined as the number of knee replacement procedures (both primary and revision) performed by the operating surgeon or hospital during the 12 months prior to the index procedure in order to reflect the level of experience of the provider at the time when patients received healthcare services [29]. In addition to surgeon and hospital volumes and 30-day readmission, patient-, physician-, and hospital-level data were also collected. Patient-level variables included age, gender, low-income status, Deyo′s Charlson Comorbidity Index (CCI), congestive heart failure (CHF) status, diabetes mellitus (DM) status, obesity status, renal failure status, and intensive care unit (ICU) admission; physician-level variables included age, orthopedic surgeon, and seniority; and hospital-level variables included accreditation status, teaching status, and geographic location.

### Cutoff point selection

Due to the potential for the relationships between surgeon and hospital volumes, respectively, and 30-day readmission to be non-linear, restricted cubic splines regression with five knots [33] was applied to model the relationship between provider volume and the risk of the provider-level, risk-adjusted 30-day readmission rate (transformed by logarithm) in order to identify any inflection point that could be used to distinguish the service volume into categories after adjusting for the abovementioned patient, surgeon, and hospital variables.

### Statistical analysis

All of the statistical analyses of the volume-outcome relationship were performed using SAS (version 9.4, SAS Institution Inc., Cary, NC, USA). In the statistical tests that were conducted in this study, a two-sided p value ≤ 0.05 was considered statistically significant. The distributional properties of continuous variables were expressed as mean ± standard deviation (SD), whereas categorical variables were represented as frequency and percentage. In univariate analysis, the potential three-level predictors of 30-day readmission were examined using a chi-square test or two-sample t-test, as appropriate. Next, in order to account for the correlations between physician (level-2) and hospital (level-3), a multivariate analysis to estimate the effects of three-level predictors on the probability of 30-day readmission was conducted by fitting mixed-effects logistic regression models to the data of each patient.

## Results

The 30,828 patients whose data were included in this study received surgeries from 1,468 surgeons in 437 hospitals. The descriptive analysis (Table 1) showed that the mean age of patients was 69.96 years and that the majority of patients were female (75.44%). Only 0.47% of patients were identified as low income. Over two-thirds (19,733; 64.01%) of patients scored a 1 or less on the Charlson comorbidity index, with the remainder scoring 2 points or higher. In terms of comorbidity, the numbers of patients who had CHF, DM, obesity, and renal failure were 1,798 (5.83%), 7,013 (22.75%), 15 (0.05%), and 667 (2.16%), respectively. Only 0.81% of patients were admitted to the ICU during hospitalization. As for surgeon characteristics, the mean age of surgeons was 48.41 years, around three-quarters of the surgeons were orthopedic surgeons, and the average seniority was 7.70 years. Slightly over one-third (38.33%) of patients received their surgeries in medical centers, with rest receiving their surgeries in regional hospitals (30.82%) and community hospitals (30.85%). Three-quarter (75.36%) of the patients received their surgery in a teaching hospital and 45.40% received their surgery in hospitals located in northern Taiwan (Taipei and Northern divisions). The average hospital and surgeon volumes were 74.35 and 22.51, respectively. A total of 1,267 patients were readmitted within 30 days of discharge, yielding a 30-day readmission rate of 4.11%.

**Table 1:** Descriptive analysis (N = 30,828).

Variable	
30-day readmission, n (%)	1,267 (4.11)
**Patient characteristics**	
Age, mean (S.D)	69.96 (8.32)
Gender (Male), n (%)	7,571 (24.56)
Low income, n (%)	144 (0.47)
Charlson Comorbidity Index, n (%)	
0	10,146 (32.91)
1	9,587 (31.10)
2	5,513 (17.88)
>2	5,582 (18.11)
CHF, n (%)	1,798 (5.83)
DM, n (%)	7,013 (22.75)
Obesity, n (%)	15 (0.05)
RF, n (%)	667 (2.16)
ICU admission, n (%)	249 (0.81)
**Surgeon characteristics**	
Average annual service volume, mean (S.D)	22.51 (50.88)
Age, mean (S.D)	48.41 (7.50)
Orthopedic surgeon, n (%)	23,243 (75.40)
Seniority, mean (S.D)	7.70 (6.01)
**Hospital characteristics**	
Average annual service volume, mean (S.D)	74.35 (139.48)
Accreditation Level	
Medical Center, n (%)	11,817 (38.33)
Regional Hospital, n (%)	9,500 (30.82)
Community Hospital, n (%)	9,511 (30.85)
Teaching status, n (%)	23,233 (75.36)
Geographic location	
Taipei division, n (%)	9,739 (31.59)
Northern division, n (%)	4,258 (13.81)
Central division, n (%)	5,148 (16.70)
Southern division, n (%)	4,939 (16.02)
Kaoping division, n (%)	6,048 (19.62)
Eastern division, n (%)	696 (2.26)

CHF: Congestive heart failure; DM: diabetes mellitus; RF: Renal failure; ICU: Intensive Care Unit

**Fig 1:**
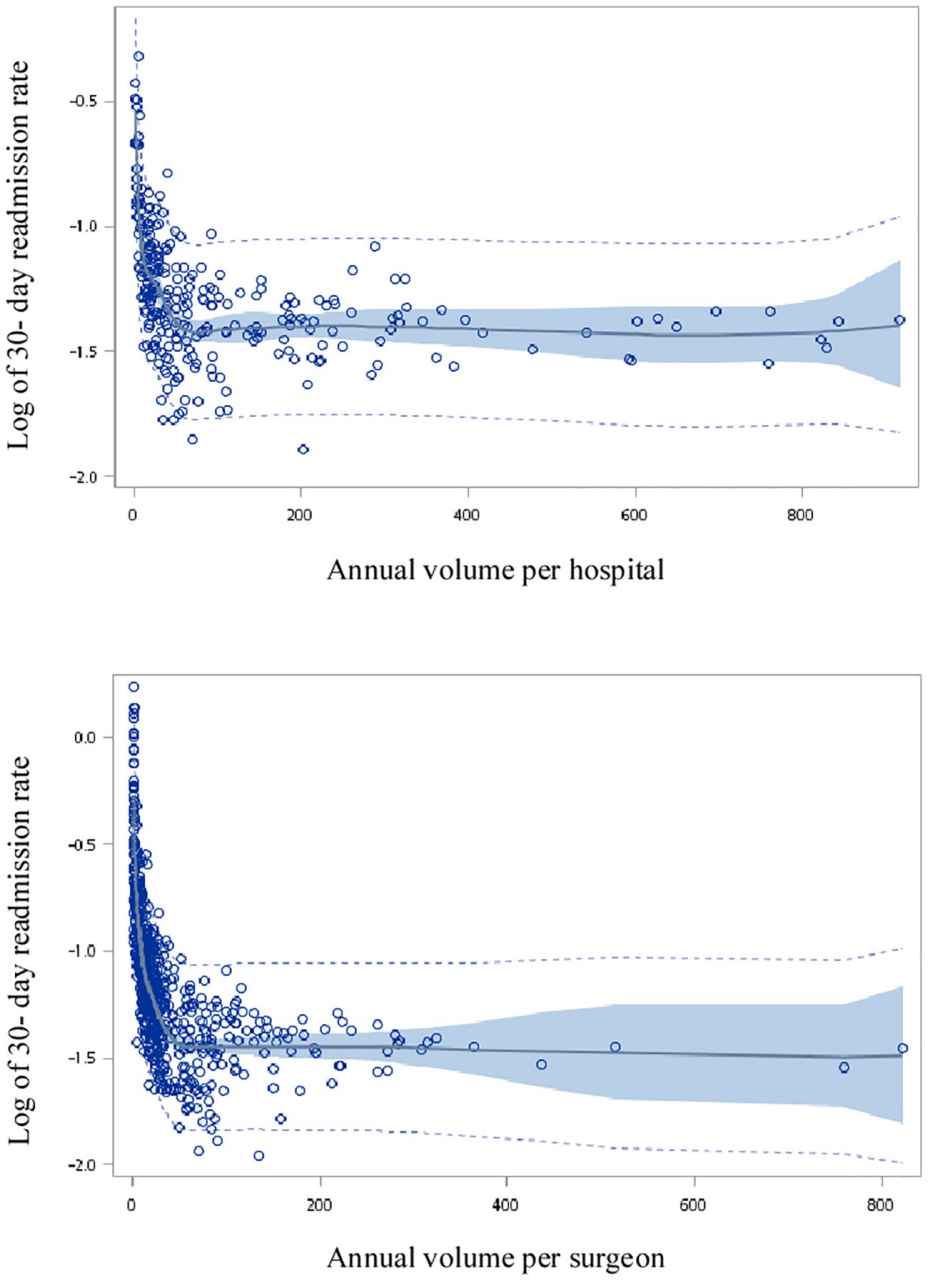
Restricted cubic spline plot of the adjusted log of the 30-day readmission rate versus annual total knee replacement volume performed per hospital and per surgeon. Note: The light dotted curve presents the 95% confidence intervals for the predicted limits. The dark line presents the fit regression model. The dark area presents the 95% confidence intervals for the regression limits.

The results of restricted cubic splines regression showed surgeon volume as inversely associated with the surgeon-level log of risk-adjusted 30-day readmission rates. However, this relationship was not linear. The optimum cutoff points of surgeon and hospital volumes were 50 and 75 procedures a year, respectively because the slopes were changed dramatically (Fig 1). Based on the results of the restricted cubic splines regression model, this study identified optimal volume standards of 50 for surgeons and 75 for hospitals.

Based on these cutoff points, 42.16% of patients received their surgeries from low volume surgeons. Patients whose surgeon was in the low volume group were slightly older (70.08 vs. 69.87, p-value = 0.029), more likely to be men (26.95% vs. 22.81%; p-value<0.001), more likely to come from a low-income household (0.72% vs 0.29%; p-value<0.001), and more likely to have comorbidities and pre-existing CHF, DM, and renal failure. In addition, this group faced a higher rate of ICU admission during their index hospitalization. The mean age and seniority of the surgeons in low volume group were, respectively, younger (45.36 vs. 50.63; p-value<0.001) and lower (7.17 vs. 8.08; p-value<0.001) and the proportion of orthopedic surgeons was higher (81.52 vs. 70.93; p-value<0.001). Moreover, the patients of surgeons who were in the low-volume group were more likely to live in central and eastern Taiwan, more likely to receive their surgeries in regional hospitals, and slightly more likely to receive their surgeries in teaching hospitals (76.59 vs. 74.47; p-value<0.001). Finally, the regional location of hospitals varied among these two groups as well (Table 2).

**Table 2:** Characteristics comparison between high-volume and low-volume surgeons.

	Low volume (<50) N = 12,996 (42.16%)	High volume (≧50) N = 17,832 (57.84%)	p-value
30-day readmission, n (%)	677 (5.21)	590 (3.31)	<0.001^¶^
**Patient characteristics**			
Age, mean (S.D)	70.08 (8.57)	69.87 (8.12)	0.029^§^
Gender			
Male, n (%)	3,503 (26.95)	4,068 (22.81)	<0.001^¶^
Female, n (%)	9,493 (73.05)	1,3764 (77.19)	
Low income, n (%)	93 (0.72)	51 (0.29)	<0.001^¶^
Charlson Comorbidity Index			
0, n (%)	4,085 (31.43)	6,061 (33.99)	<0.001^¶^
1, n (%)	4,053 (31.19)	5,534 (31.03)	
2, n (%)	2,342 (18.02)	3,171 (17.78)	
>2, n (%)	2,516 (19.36)	3,066 (17.19)	
CHF, n (%)	819 (6.30)	979 (5.49)	0.003^¶^
DM, n (%)	3,029 (23.31)	3,984 (22.34)	0.046^¶^
Obesity, n (%)	5 (0.04)	10 (0.06)	0.489^¶^
RF, n (%)	323 (2.49)	344 (1.93)	0.001^¶^
ICU admission, n (%)	154 (1.18)	95 (0.53)	<0.001^¶^
**Surgeon characteristics**			
Age, mean (SD)	45.36 (7.50)	50.63 (6.68)	<0.001^§^
Seniority, mean (SD)	7.17 (5.23)	8.08 (6.50)	<0.001^§^
Orthopedic surgeon, n (%)	10,594 (81.52)	12,649 (70.93)	<0.001^§^
**Hospital characteristics**			
Accreditation Level			<0.001^¶^
Medical Center, n (%)	3,531 (27.17)	8,286 (46.47)	
Regional Hospital, n (%)	5,718 (44.00)	3,782 (21.21)	
Community Hospital, n (%)	3,747 (28.83)	5,764 (32.32)	
Teaching status (yes), n (%)	9,953 (76.59)	13,280 (74.47)	<0.001^¶^
Location			<0.001^¶^
Taipei division	3,643 (28.03)	6,096 (34.19)	
Northern division	2,034 (15.65)	2,224 (12.47)	
Central division	2,887 (22.21)	2,261 (12.68)	
Southern division	1,691 (13.01)	3,248 (18.21)	
Kaoping division	2,291 (17.63)	3,757 (21.07)	
Eastern division	450 (3.46)	246 (1.38)	

^¶^ χ^2^ test

^§^ t-test

CHF: Congestive heart failure; DM: diabetes mellitus; RF: Renal failure; ICU: Intensive Care Unit

Table 3 compares the characteristics of low-volume and high-volume hospitals. One-fifth (21.36%) of the patients received their surgeries in low-volume hospitals. The differences of patient-, surgeon-, and hospital-level characteristics between high- and low-volume hospitals were largely the same as the differences between high- and low-volume surgeons, with the notable exceptions of patient age, pre-existing DM, and renal failure.

**Table 3:** Characteristics comparison between high-volume and low-volume hospitals.

	Low volume (<75) N = 6,585 (21.36%)	High volume (≧75) N = 24,243 (78.64%)	p-value
30-day readmission, n (%)	355 (5.39)	912 (3.76)	<0.001^¶^
**Patient characteristics**			
Age, mean (SD)	70.04 (8.38)	69.94 (8.30)	0.387
Gender			
Male, n (%)	1,760 (26.73)	5,811 (23.97)	<0.001^¶^
Female, n (%)	4,825 (73.27)	18,432 (76.03)	
Low income, n (%)	49(0.74)	96 (0.40)	<0.001^¶^
Charlson Comorbidity Index			0.010^¶^
0, n (%)	2,062 (31.31)	8,084 (33.35)	
1, n (%)	2,058 (31.25)	7,529 (31.06)	
2, n (%)	1,225 (18.60)	4,288 (17.69)	
>2, n (%)	1,240 (18.83)	4,342 (17.91)	
CHF, n (%)	428 (6.50)	1,370 (5.65)	0.009^¶^
DM, n (%)	1,498 (22.75)	5,515 (22.75)	1.000^¶^
Obesity, n (%)	3,(0.05)	12,(0.05)	0.898^¶^
RF, n (%)	147 (2.23)	520 (2.14)	0.666^¶^
ICU admission, n (%)	100 (1.52)	149 (0.61)	<0.001^¶^
**Surgeon characteristics**			
Age, mean (SD)	46.60 (7.36)	48.90 (7.47)	<0.001
Orthopedic surgeon, n (%)	5,274 (80.09)	17,969 (74.12)	<0.001
Seniority, mean (SD)	6.84 (4.91)	7.93 (6.26)	<0.001
**Hospital characteristics**			
Accreditation Level			<0.001^¶^
Medical Center, n (%)	141 (2.14)	11,676 (48.16)	
Regional Hospital, n (%)	3,079 (46.76)	6,421 (26.49)	
Community Hospital, n (%)	3,365 (51.10)	6,146 (25.35)	
Teaching status (yes), n (%)	3,836 (58.25)	19,397 (80.01)	<0.001^¶^
Location			<0.001^¶^
Taipei division	1,429 (21.70)	8,310 (34.28)	
Northern division	1,202 (18.25)	3,056 (12.61)	
Central division	1,314 (19.95)	3,834 (15.81)	
Southern division	789 (11.98)	4,150 (17.12)	
Kaoping division	1,580 (23.99)	4,468 (18.43)	
Eastern division	271 (4.12)	425 (1.75)	

^¶^ χ^2^ test

^§^ t-test

CHF: Congestive heart failure; DM: diabetes mellitus; RF: Renal failure; ICU: Intensive Care Unit

Finally, Table 4 shows the results of the multilevel logistic regression model, which demonstrate that patients who received their surgeries from a low-volume surgeon faced a higher risk of 30-day readmission after discharge, after adjusting for patient-, surgeon-, and hospital-level covariates. In addition to surgeon volume, the results also revealed that patient age, patient gender, Charlson Comorbidity Index score, DM status, obesity status, renal failure status, ICU admission, and surgeon seniority were each risk factors as well.

**Table 4:** Results of multilevel logistic regression.

	OR	95%C.I.	p-value
**Patient characteristics**			
Age	1.01	1.00–1.02	0.015
Gender (ref = female)	1.48	1.31–1.67	<0.001
Low income (ref = no)	1.79	0.99–3.21	0.053
Charlson Comorbidity Index (ref = 0)			
1	1.31	1.12–1.55	0.001
2	1.51	1.26–1.81	<0.001
>2	1.91	1.58–2.30	<0.001
CHF (ref = no)	1.18	0.96–1.46	0.118
DM (ref = no)	1.17	1.02–1.34	0.028
Obesity (ref = no)	4.34	1.16–16.18	0.029
RF (ref = no)	1.83	1.39–2.40	<0.001
ICU admission (ref = no)	2.30	1.53–3.46	<0.001
**Surgeon characteristics**			
Volume (ref = HV)	1.44	1.22–1.69	<0.001
Age	1.00	0.99–1.01	0.929
Orthopedic surgeon (ref = no)	0.95	0.82–1.11	0.545
Seniority	0.98	0.96–0.99	0.002
**Hospital characteristics**			
Low volume (ref = high)	1.07	0.90–1.28	0.435
Accreditation Level (ref = community Hospital)			
Medical Center	1.04	0.80–1.35	0.753
Regional Hospital	0.86	0.69–1.07	0.174
Teaching status (ref = no)	0.85	0.68–1.06	0.144
Location (ref = Taipei division)			
Northern division	1.05	0.84–1.32	0.649
Central division	1.12	0.91–1.38	0.275
Southern division	1.15	0.92–1.43	0.220
Kaoping division	1.09	0.88–1.36	0.413
Eastern division	1.56	1.08–2.25	0.018

OR: Odds ratio; C.I.: Confidence Intervals

CHF: Congestive heart failure; DM: diabetes mellitus; RF: Renal failure; ICU: Intensive Care Unit

## Discussion

Thresholds of 50 cases a year for surgeons and 75 cases a year for hospitals were identified. However, only the surgeon volume threshold was associated with TKA 30-day readmission.

The surgeon and hospital volume thresholds for TKA were identified using a restricted cubic spline regression model. The effects of cutoff point selection on the study of volume-outcome issues have been discussed, with many studies indicating that this may lead to disparate and controversial findings. In addition, existing studies typically assumed the relationship between volume and outcome to be linear. Moreover, the most common method of categorization used in these studies, the percentile method (e.g., quartile, tertile), is also built upon the assumption of a normal distribution of provider volume. Thus, the appropriateness of existing categorization methods should be reviewed if this assumption is not valid [3, 4]. Several limitations of categorizing quantitative measurements, including loss of information and reduction in power, have been described previously [34]. The appropriate application of categorization may be one of the most important current issues in the field of volume-outcome studies [27]. Most recently, studies on the volume-outcome relationship have adopted spline function methods such as restricted cubic spline functions [29, 35, 36] and the locally weighted scatterplot smoothing (LOESS) method [37, 38]. The categorization method used in this study allows researchers to visualize the cutoff point in order to determine the optimal volume threshold.

This study found that physician volume rather than hospital volume was associated with TKA outcomes, which is similar to Wei et al [22] and different from Manley et al [39], Paterson et al [16] (only hospital volume was significant), and Bozic et al (both surgeon and hospital volumes were significant) [30]. Except for Manley et al, the other studies used quartile to categorize service volume and to define the low-volume group. Furthermore, the outcome variables among these studies differed. Manley et al focused on revision; Patersen *et al* focused on complications, 90-day mortality, 1-year readmission, and revision; and Bozic *et al* focused on mortality, 30-day readmission and reoperation, complications, and length of stay. Finally, the definition of service volume used in this study differed significantly from the definitions used in prior research. Thus, directly comparing the results of this study to those of previous studies may not be appropriate.

“Practice makes perfect” and “selective referral” are the two primary hypotheses as to why a relationship exists between volume and outcome in TKA [40]. The former presumes that surgeons who perform more (higher volumes) of a particular procedure become increasingly proficient in that procedure. The latter postulates that patients are referred to hospitals and physicians, respectively, based on their track records of better outcomes. The findings of this study seem to support the “practice makes perfect” hypothesis over the “selective referral” hypothesis. Lin et al suggested that if the hospital volume effect is not statistically significant when physician volume is added into the statistical model, then the practice makes perfect hypothesis is sustained [41]. Further, the Taiwan government has operated the NHI program since 1995, which today covers more than 99% of the population and gives people complete freedom of choice among providers. Centralization has also not been implemented for TKA in Taiwan. High volume may serve as a proxy for other factors such as experience and skill. In addition, high-volume surgeons may be more familiar with treatment guidelines and newer techniques and be better able to treat related complications [42].

The strength of this study was that a population-based study was conducted and a restricted cubic spline model was applied to determine an optimal volume threshold to account for the non-linear relationship between volume and outcomes. This study was potentially affected by several limitations. Firstly, the cutoff point of service volume per year may not be generalizable beyond the studied setting. Future research may adopt the technique used in this study to identify appropriate volume thresholds in other settings. Secondly, due to the limitation of retrospective study design in which not all information can be obtained, although claims data offer a significant amount of clinical information, potential confounders that we were unable to adjust for include body mass index, smoking status, duration of operation, guideline adherence, and American Society of Anesthesiologists (ASA) grade, among others.

## Conclusion

In this study, according to the results of the restricted cubic spline models, the optimal volume thresholds for surgeons and hospitals are 50 cases and 75 cases a year, respectively. However, only the surgeon volume threshold is associated with 30-day readmission. The optimal volume is not a difficult bar to achieve. Policy makers may consider regularly publishing operating volumes or setting the optimal volume levels for surgeons and hospitals. Further, it may be possible to provide service-volume-related information to help guide patients to select experienced providers. In addition, setting the optimal volume as a criterion of recertification may be a feasible method to ensure the quality of TKA surgeries.
